# Recruitment of Informal Caregivers of Persons with Dementia into Clinical Trial: The power of the Messenger.

**DOI:** 10.1093/geroni/igaf122.2230

**Published:** 2025-12-31

**Authors:** Zayn Boustani, Mariana Stavig, Hanani Ndebele, Jordan Hill, Miriam Jocelyn Rodriguez, Lilian Golzarri-Arroyo, Richard Holden, Malaz Boustani

**Affiliations:** Indiana University Bloomington, Chicago, Illinois, United States; Indiana University Bloomington, Bloomington, Indiana, United States; Indiana University Bloomington, Bloomington, Indiana, United States; Indiana University Bloomington, Bloomington, Indiana, United States; Indiana University Bloomington, Bloomington, Indiana, United States; Indiana University Bloomington, Bloomington, Indiana, United States; Indiana University Bloomington, Bloomington, Indiana, United States; Indiana University, Indianapolis, Indiana, United States

## Abstract

**Intro:**

The success of a randomized controlled trial depends on the timely and effective recruitment of participants. We aimed to investigate the effect of a trusted messenger on the recruitment process into a randomized controlled trial testing the efficacy of a mobile caregiver intervention in reducing caregiver burden and patient behavioral and psychological symptoms of dementia (BPSD).

**Methods:**

A total of n = 4630 potential participants were contacted. Recruitment occurred via (1) “Cold Call,” where patients from a health system registry were called by research staff, and (2) “trusted messenger,” where research staff called patients who were referred directly from a memory care physician. The outcome measure was the call-to-consent ratio. A two-population proportion Z-test compared the ratios between the two recruitment methods.

**Results:**

The enrollment rate was 23.88% for participants recruited via the “trusted messenger” method and 1.18% for those recruited from the “cold calls” method (z = 15.11, p ≈ 0.0000).

**Conclusions:**

“Trusted Messenger” was 20 times more effective than “Cold Calls” in recruiting human subjects into a clinical trial. Trusted Messenger leverage preexisting relationship. Although “Cold calls” is perceived as easy for the researcher to complete, it yields fewer enrollments per points of contact. Future studies should examine and compare other recruitment methods among ethnically diverse samples such as community outreach, flyer posting, and door-to-door solicitation.

